# The Hedgehog Pathway Conditions the Bone Microenvironment for Osteolytic Metastasis of Breast Cancer

**DOI:** 10.1155/2012/298623

**Published:** 2011-12-01

**Authors:** Shamik Das, Rajeev S. Samant, Lalita A. Shevde

**Affiliations:** Department of Oncologic Sciences, USA Mitchell Cancer Institute, Mobile, AL 36608, USA

## Abstract

The microenvironment at the site of tumor metastasis plays a key role in determining the fate of the metastasizing tumor cells. This ultimately has a direct impact on the progression of cancer. Bone is the preferred site of metastasis of breast cancer. Painful, debilitating osteolytic lesions are formed as a result of crosstalk between breast cancer cells and cells in the bone, predominantly the osteoblasts and osteoclasts. In this paper, we have discussed the temporal and spatial role of hedgehog (Hh) signaling in influencing the fate of metastatic breast cancer cells in bone. By virtue of its secreted ligands, the Hh pathway is capable of homotypic and heterotypic signaling and consequently altering the microenvironment in the bone. We also have put into perspective the therapeutic implications of using Hh inhibitors to prevent and/or treat bone metastases of breast cancer.

## 1. Introduction

The overwhelming numbers of cancer patients (≥90%) that die due to the dissemination of cancer cells rather than the primary tumor throw the process of metastasis to the centre stage of clinical management of cancer [1]. However, even as we embark on this review, the most poorly understood aspect of the pathogenesis and progression of cancer is the process of metastasis of the tumor.

**Figure 1:**
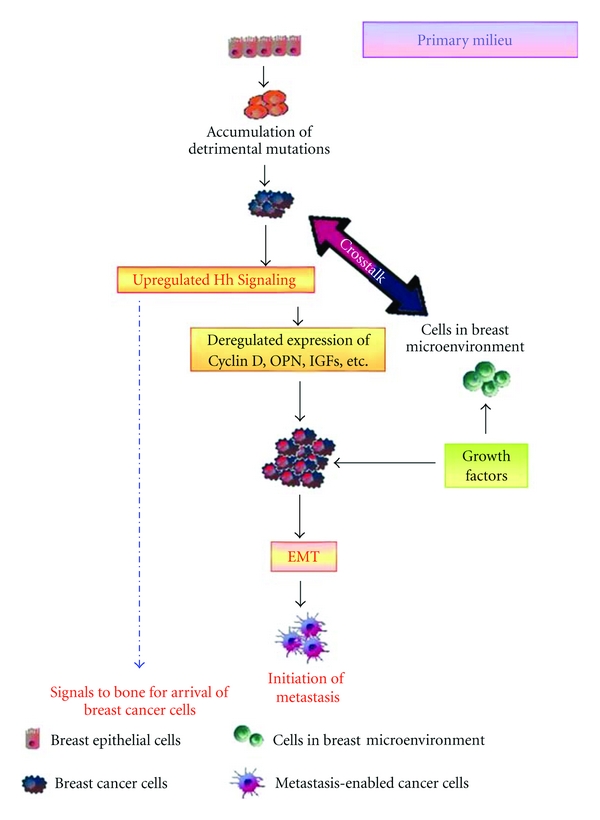
Hh signaling conditions the milieu to support metastasis of breast cancer cells to the bone. Depicted here is the first of the two microenvironments, the milieu of the primary tumor. Hh signaling in the tumor cells impacts the stromal cells in the environment, which in turn amplify paracrine Hh signaling by producing growth factors that propel epithelial-mesenchymal transition. Concomitantly, secreted, soluble proteins produced by the primary tumor contribute towards conditioning the secondary site for the arrival of the tumor cells.

**Figure 2:**
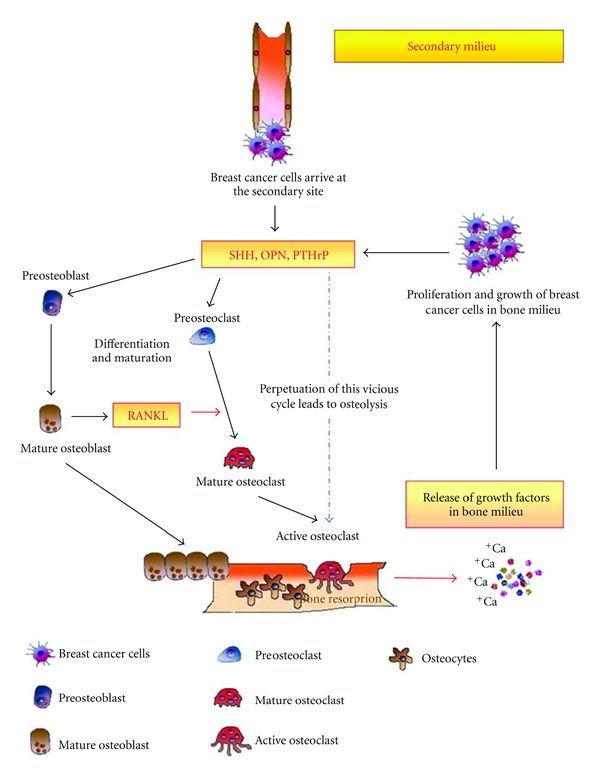
Breast cancer cells armed with Hh signaling disrupt the dynamic equilibrium in the bone to serve its purpose of self propagation and subsequent osteolysis. Breast cancer cells engane in a crosstalk with osteoblasts and osteoclasts. This cumulatively results in the differentiation and activation of osteoclasts and eventually leads to enhancing osteolysis and growth of breast tumor cells in the bone. Overall, this figure addresses the role of Hh signaling in the vicious cycle of osteolytic metastasis of breast cancer.

Evolving literature supports that metastasis is a second disease imposed on the primary tumor. The outcome of metastasis is determined by the interplay between the subpopulation of metastatic cells and host homeostatic factors in the specific organ microenvironment [2]. The metastatic cascade can be conceptually organized and simplified into two major phases: (i) physical translocation of a cancer cell from the primary tumor to the microenvironment of a distant tissue (Figure 1) and (ii) colonization of secondary site (Figure 2) [3].

The metastasizing tumor cells hijack many of the pathways that play major roles during normal development. Many of the embryonic developmental signaling pathways, such as the Wnt, Hedgehog (Hh), and Notch pathways, affect the survival of tumor stem cells and orchestrate a complex microenvironment that promotes tumor survival and progression. In this review, we will highlight the significance of the Hh pathway in developmental biology and our present understanding of its role in regulating breast cancer metastasis to bone. We will elaborate how a pathway that is so critical in normal development of the embryo is usurped by the breast cancer cells to serve their own purpose of invading the tissue of its origin, extravasation, survival during translocation, and adaptation at the distant site to bring about proliferation and colonization.

## 2. The Hh Pathway in Normal Development

The Hh pathway plays a central role in embryonic development and maintenance of stem or progenitor cells in many adult tissues [4]. The Hh family of secreted proteins signal through both autocrine and paracrine mechanisms to control cell proliferation, differentiation, and morphology [5]. The ligands comprise desert hedgehog (DHH), Indian hedgehog (IHH), and Sonic hedgehog (SHH). Hh signaling in mammalian cells is mediated by the GLI family of zinc finger transcription factors comprising GLI1, GLI2, and GLI3. GLI1 is a strong transcriptional activator; GLI2 can function as an activator or a repressor in a context-dependent manner; GLI3 is mostly a repressor [6]. In its classical form, in the absence of the ligand, the Hh-signaling pathway is inactive, GLI1 is sequestered in the cytoplasm and repressed for its transcription activity. Binding of the Hh ligands to the receptor, a 12-pass transmembrane protein called patched-1 or patched-2 (PTCH1 or -2), releases the inhibitory affect of PTCH on a serpentine protein called Smoothened (SMO) [7]. SMO gets hyperphosphorylated and localizes to primary cilia where [8] GLI1 is activated by release from a large protein complex and translocates to the nucleus to function as a transcriptional activator [9] of several target genes, including PTCH, insulin-like growth factor-binding protein and cyclin D2 [10].

The involvement of the Hh pathway, in particular the ligand SHH, with the skeletal system begins with embryonic development, where SHH is expressed in the notochord, the floorplate of the neural tube, the brain, the zone of polarizing activity in the developing limbs, and the gut [11, 12]. SHH specifically functions in many different ways to contribute to the patterning of a developing embryo in a concentration-dependent manner along a target range [13]. A variety of embryonic defects and diseases result from mutations in the Hh pathway [14]. The long-range morphogenic properties of SHH signaling are also evident in the development of the CNS [15]. Thus, temporal and spatial regulation of SHH signaling is key to proper organogenesis. However, in the adults, this pathway is mainly inactive [16] and may play a role in the maintenance and renewal of normal stem cell population in the nervous system [17]. Moreover, Lavine et al. reported that the Hh signaling is essential for cardiac function at the level of the coronary vasculature [18].

## 3. The Hedgehog Pathway in Cancer

The Hh pathway is required for normal proliferation of human melanocytes *in vitro* and for proliferation and survival of human melanoma *in vivo* [19, 20]. In esophageal squamous cell carcinoma, GLI1 expression has been associated with lymphatic metastasis [21], while in breast cancer, strong nuclear GLI staining was observed [22]. Li et al. have recently reported that pancreatic cancer stem cells express high levels of SHH [23]. This is interesting given the implications for SHH in adult stem cell renewal, in pancreatic ductal progenitor cells, and also in adult hair follicle stem cells [24]. SHH is misregulated in pancreatic adenocarcinoma, prostate adenocarcinoma, esophageal and stomach cancer, and nonsmall cell carcinoma [14]. As such, Hh signaling has been shown to be active in multiple cancer types [22, 25–48] (Table 1).

Active Hh signaling is also found to influence the tumor stromal microenvironment [27] and supports stem cells in the tumor in an undifferentiated, proliferative state [26, 50]. SHH is not only a mediator of angiogenesis but has also been shown to induce vessel formation in endothelial cells [51] and activate expression of angiopoietins I and II, and VEGF-signaling proteins from mesenchymal cells, highlighting the significance of tumor-associated fibroblasts in combination with canonical Hh signaling to mediate blood vessel formation [52]. Cancer cells utilize abnormal Hh signaling (both autocrine and paracrine) to influence proliferation and differentiation of their surrounding environment.

The role of Hh signaling in cancer has been revealed by studies that have manipulated the expression of the GLI transcription factors or the ligands or upon treatment with pharmacologic inhibitors that restrict Hh signaling. In pancreatic cancer cell lines, disruption of Hh signaling by the inhibitor cyclopamine, inhibited epithelial-mesenchymal-transition (EMT) [53, 54]. Tumor burden and metastasis in both prostate and pancreatic adenocarinomas were also reduced as a result of Hh signaling inhibition [53, 55]. In contrast, enforced expression of GLI1 induced the expression of Snail [56], an EMT marker. Conversely, we observed loss of mesenchymal markers upon abrogation of GLI1 expression [19]. Overall, GLI1 silencing had a pronounced effect on tumor malignancy *in vivo *by reducing metastasis. We also reported that signaling via the Hh pathway transcriptionally upregulates OPN [19]. OPN is a secreted protein that influences multiple downstream signaling events that allow cancer cells to resist apoptosis, invade through extracellular matrix, evade host immunity [57], and influence growth of indolent tumors [58, 59]. OPN constitutes a component of the secretome of several melanoma-derived cell lines [60, 61] and is also expressed in metastatic breast cancer cell lines [62]. It is highly probable that active Hh signaling in a subset of cancer cells can be propagated in a paracrine manner by OPN secreted into the tumor microenvironment. OPN, by virtue of its ability to signal through multiple receptors, can promote malignant behavior in neighboring cancer cells, regardless of the status of the Hh pathway, thereby propagating paracrine Hh signaling. Thus, at the site of origin, the breast tumor cells not only potentiate their own aggressiveness by influencing the neighboring cells, but also send signals to the secondary target organ to condition for relocalization [58, 63, 64].

For the purpose of this review, we have focused the remainder of the article on discussing the role of Hh signaling in impacting breast cancer metastasis to the bone. This complication of breast cancer continues to present a challenge to oncologists and reduces the chances of survival for breast cancer patients. Among breast cancers that become aggressive, metastasis to bone marrow is common. Detection of bone metastasis often signals the onset of the life-threatening phase of breast cancer. The 5-year survival rate is 98% for breast cancer when detected early; this precipitously drops to 83% for patients initially diagnosed with regional spread and to 26% for those with distant metastases. In the following sections, we will discuss the role of Hh signaling in mediating a crosstalk between breast cancer cells and cells in the bone and the overall impact on the ability of breast cancer cells to sculpt the bone microenvironment and cause osteolysis (Figures 1 and 2).

## 4. The Bone Microenvironment

The bone microenvironment comprises osteoblasts, osteoclasts, mineralized bone matrix, and other cell types, such as the osteocytes embedded within bone. Of these, the most important ones (from the perspective of this article) are the bone-resorbing osteoclasts and bone-forming osteoblasts.

Osteoblasts are derived from mesenchymal stem cells, which can also give rise to chondrocytes, fibroblasts, myocytes, or adipocytes [65]. Formation of new bone and the regulation of osteoclastogenesis through expression of RANKL and OPG are two main functions of the osteoblasts. Various growth factors and hormones like BMPs, PTHrP, TGF0*β*, and so forth are known to take part in the differentiation of preosteoblasts into mature osteoblasts. Eventually, mature, mineralizing osteoblasts become embedded in the newly secreted bone matrix and undergo terminal differentiation to form osteocytes. Although the osteocytes have much reduced activity as compared to osteoblasts, their long processes allow them to connect the entire matrix via a series of canaliculi. It is understood that the osteocytes ensure communication between sites deep in the bone and the extraosseous world; they create an enormous increase in mineral surface exposed to extracellular fluid and cellular activity and function as mechanosensory cells of bone, involved in the transduction of mechanical loads into biochemical signals [66].

Osteoclasts, on the other hand, are large multinucleated terminally differentiated cells with a unique ability for bone resorption [67]. They are derived from hematopoietic stem cells. The cells undergo proliferation in response to M-CSF. The precursor cells flaunt receptor activator of nuclear factor *κ*B (RANK) on the surface, while the ligand RANKL is expressed by the bone marrow stromal cells and osteoblasts. Binding of the ligand to the receptor commits the precursor cells to the osteoclast lineage. The same interaction is also critical for osteoclast formation and can also promote osteoclast activity, since RANK is also present on the surface of terminally differentiated osteoclasts. The fusion of osteoclast precursor cells results in the formation of large multinucleated active osteoclasts.

Osteoprotegerin (OPG) is a soluble decoy receptor and a competitor of RANKL in its binding with RANK and thus can inhibit osteoclastogenesis. Therefore, the balance of RANKL and OPG is critical for osteoclast formation and activity. Osteoclasts attach to the bone surface via actin-rich podosomes enabling them to form sealed zones with ruffled borders. Proteolytic enzymes such as CTSK (Cathepsin K) and MMPs are secreted into this isolated environment, resulting in degradation of the bone matrix, dissolution of the bone mineral, and resorption of the bone [68]. Evidently behind its outward rigidity, bone is a highly dynamic organ where homeostasis is tightly controlled and largely dependent upon cellular communication between osteoclasts and osteoblasts. This tight coupling between bone resorption and bone formation is essential for the correct function and maintenance of the skeletal system, repairing microscopic skeletal damage, and replacing aged bone. Any deviation from this homeostasis results in a range of pathologic diseases, including osteoporosis and cancer-induced bone disease.

## 5. The Metastasis of Breast Cancer Cells to the Bone

The vertebral venous system is the most common mode of transport of breast cancer cells from the breast to bone [69]. This allows breast cancer cells to come into contact with the axial skeleton, including the ribs, spine, pelvis, and proximal humerus and femur, which is the main distribution of bone metastases in breast cancer patients [70]. Tumor cells, even at their site of origin, send signals to their preferred secondary site [64] of metastasis. This modulates the micro-environment of that region. It is likely that the Hh ligands and secreted factors such as IGFs and OPN may impact this “homing” mechanism. It can be speculated that the factors secreted by breast cancer cells create a “premetastatic niche” as termed by Lyden and colleagues [64, 71]. The role of chemokines and cytokines as well as the homing mechanism has also been elaborately discussed in a review by Bussard et al. [72]. Our findings show that expression and secretion of Hh ligands by the breast cancer cells augments these processes (Figure 1). Once malignant cells have migrated to the bone, their ability to colonize is facilitated by the bone microenvironment. MMPs, chemokine receptor 4 (CXCR4), VEGF, and connective tissue growth factors supposedly target metastatic tumor cells to bone and facilitate their survival within the bone microenvironment [73, 74]. Physical factors within the bone microenvironment, including hypoxia, acidic pH, and extracellular calcium, and bone-derived growth factors, such as TGF-*β* and insulin-like growth factors activate tumor expression of VEGF, PDGF, and endothelin (ET-1) [75]. Factors such as PTHrP, TGF-*β*, and IL-11 produced by breast cancer cells favor osteoclast maturation and osteolysis, leading to the release of growth factors that stimulate malignant tumor growth [76]. In fact, expression of IL-11 and OPN by breast cancer cells has been found to be critical for the osteolytic activity of breast cancer cells [74]. Thus, signals from the breast cancer cells at their primary site might trigger a cascade of events involving the osteoblast-mediated initiation of osteoclastogenesis which releases a plethora of growth factors in the bone milieu which not may only act as chemoattractants for the “metastasis-enabled” breast cancer cells but also favor the latter's establishment and further proliferation once they have migrated to the bone. This would in turn tilt the balance in favor of osteoclastogenesis as more favorable factors are then readily available to the osteoclasts in the bone milieu itself and thus would lead to a self-perpetuating *vicious cycle* of events (Figure 2).

## 6. Hh Signaling in the Bone Microenvironment

Hh-signaling-activated GLI2 transcription mediates osteoblast differentiation [77]. This is likely due to the regulated expression of bone morphogenetic protein-2, BMP-2, that is involved in osteogenic differentiation by promoting commitment of mesenchymal stem cells to the osteoblast lineage. GLI2 transcriptionally activates BMP-2 expression and also synergizes with BMP-2 in osteoblasts [78]. These contentions are contradicted by Plaisant et al. who have reported that Hh signaling causes a decrease in the expression of Runx2, a key transcription factor that regulates osteoblast differentiation [79]. It is proposed that Hh signaling may be regulating different aspects of bone formation in rodent and human systems.

OPN is one of the abundant noncollagenous proteins in bone. It promotes osteoclast function and is consistently overexpressed in highly metastatic cells. OPN accumulates at cement lines in remodeling bone [80] and is localized to cell-matrix and matrix-matrix interfaces in mineralized tissue, where it is deposited by actively resorbing osteoclasts. OPN positively impacts osteoclast formation, migration, and resorptive activity [81, 82]. We recently reported that OPN is regulated, in part, by the Hh pathway [19]. We have also shown that breast cancer cells express Hh ligands and engage in a crosstalk with osteoblasts and osteoclasts [83]. Our recent studies (communicated to Breast Cancer Research) have shown that the Hh pathway plays a role in initial osteoblasts maturation, especially in the presence of breast cancer cells (Figure 2). Following an initial accelerated differentiation process, characterized by the expression of alkaline phosphatase and expression of collagenous and noncollagenous matrix proteins such as BSP and OPN and osteoclast-maturation proteins including RANKL and PTHrP, the osteoblasts appear to undergo apoptosis.

The Hh ligands also mediate a direct dialogue between breast cancer cells and preosteoclasts and induce changes in preosteoclasts that influence the production of OPN and essential bone-resorbing proteases, CTSK, and MMP9 by osteoclasts [83]. Thus, Hh ligands produced by the metastasizing breast cancer cells are instrumental in initiating a crosstalk directly with osteoclasts and promote osteoclast differentiation and resorption activity (Figure 2). Breast cancer cells also express PTHrP as a result of Hh signaling, further amplify paracrine Hh signaling in the bone microenvironment, and add to the overall osteolytic conditions [84].

Thus, the vicious cycle of bone metastasis involves a complex crosstalk between the metastasizing breast tumor cells and the bone microenvironment through multiple extracellular factors and signaling pathways with the Hh pathway playing an essential role. Based on our findings, we would like to propose that the newly arrived breast tumor cells induce initial osteoblast differentiation which stimulates osteoclast differentiation. Soon, the situation is overwhelmed by osteoclast differentiation followed by intense bone resorption leading to the local release of generous amounts of growth factors that not only encourage their growth but also alter their phenotype, making them (cancer cells) resistant to standard cytotoxic antitumor treatments see the appendix [85, 86].

## 7. Conclusion

The bone microenvironment with ongoing bone resorption almost resembles sites of wound healing. The bone stroma is almost guaranteed to provide hospitable sites for disseminating colonization-competent breast cancer cells [61]. This ensures the successful proliferation and ultimate colonization of the bone by metastasizing breast tumor cells. The crosstalk between the metastasizing breast cancer cells and the bone cells, namely, the osteoblasts and the osteoclasts occurs in a fashion that not only favors proliferation of the newly arrived tumor cells in the bone milieu but also ultimately the complete subjugation of the resident (bone) pathways to serve the purpose of establishment and well-being of the tumor cells with concurrent destruction of the host environment. Therefore, it is essential to understand the interactions between tumor and bone and identify microenvironment-selective agents to halt tumor growth and bone metastasis thereby reducing the morbidity of skeletal related events [62]. Thus, given the fact that breast cancer cells express Hh ligands and that Hh signaling propels breast cancer progression, it is likely that administration of pharmacological Hh inhibitors can inhibit Hh signaling in both breast cancer cells and osteoclasts and may reduce breast-cancer-mediated bone loss in metastatic disease. This strategy targets the tumor cells as well as the bone and its microenvironment and can reduce tumor burden and tumor-derived bone lesions. 

## Figures and Tables

**Table 1:** Cancers with aberrant activation of Hh signaling.

Milieu	Hh Signaling caused by	Molecule(s) involved	Type of cancer	Reference
I	Overexpression	GLI1	Glioblastoma	[30]
	Mutations	PTCH	Basal cell carcinoma (BCC)	[31, 32]
		SMO	Basal cell carcinoma	[31, 32]
		PTCH	Medulloblastoma	[33]
		PTCH	Rhabdomyosarcoma	[34]
		PTCH1	Gorlin syndrome BCC	[35, 36]
		SMO & PTCH1	Nonfamilial BCC	[37]

II	Ligand-dependent autocrine		Breast	[22]
			Pancreatic	[38]
			Lung cancer	[39]
			Oesophagal	[40]
			Prostate	[41]
			Gastric adenocarcinoma	[42]
			Colorectal	[43]
			Hepatocellular adenocarcinoma	[44]
			Ovarian carcinoma	[45, 49]
	Ligand-dependent paracrine		Pancreatic	[46–48]

Milieu I represents the microenvironment of the primary tumor; Milieu II represents the microenvironment at the metastatic site.

## Appendix

*Some of the Key Players in Osteolytic Metastasis of Breast Cancer*

BMP: bone morphogenetic protein, a group of cytokines responsible for the tissue architecture throughout the body.
IGF: insulin-like growth factors are responsible for cell proliferation and form the IGF axis.
PDGF: platelet derived growth factor, a secreted molecule that regulates growth and cell division.
PTHrP: parathyroid hormone-related protein is a hormone that regulates endochondral bone development and also regulates epithelial mesenchymal interactions in mammary gland formation. It is secreted by several cancer cells.
MMPs: matrix metalloproteases are zinc-dependent endopeptidases, capable of degrading all kinds of extracellular matrix proteins and processing a number of bioactive molecules. They play a major role on cell proliferation, migration (adhesion/dispersion), differentiation, angiogenesis, apoptosis, and host defense.
OPG: osteoprotegerin (OPG), also known as osteoclastogenesis inhibitory factor (OCIF), or tumor necrosis factor receptor superfamily member 11B (TNFRSF11B), is a basic glycoprotein that is a decoy receptor for the receptor activator of nuclear factor kappa B ligand (RANKL) and can inhibit osteoclastogenesis.
RANK: receptor activator of nuclear factor *κ*B (RANK), also known as TRANCE Receptor, is a type I membrane protein expressed on the surface of osteoclasts and is involved in their activation upon ligand binding.
RANKL: receptor activator of nuclear factor kappa B ligand, also known as tumor necrosis factor ligand superfamily member 11 (TNFSF11), TNF-related activation-induced cytokine (TRANCE), osteoprotegerin ligand (OPGL), and osteoclast differentiation factor (ODF). It functions as a key factor for osteoclast differentiation and activation.
TGF-β: transforming growth factor beta is an antiproliferative factor protein that controls proliferation, cellular differentiation, and other functions in most cells.
VEGF: vascular endothelial growth factor is a signal protein produced by cells that stimulates vasculogenesis and angiogenesis.

## Abbreviations

BMP: Bone morphogenetic protein
CTSK: Cathepsin K
CXCR4: Chemokine receptor 1
DHH: Desert hedgehog
EMT: Epithelial-Mesenchymal transition
ET-1: Endothelin-1
GLI: Glioma-associated oncogene
Hh: Hh pathway
IHH: Indian Hedgehog
IL-11: Interleukin-11
M-CSF: Macrophage colony-stimulating factor
MMP9: Matrix metalloprotease 9
OPG: Osteoprotegerin
OPN: Osteopontin
PTCH: Patched
PDGF: Platelet-derived growth factor
PTHrP: Parathyroid Hormone-related protein
RANK: Receptor activator of NF-*κ*B
RANKL: Receptor activator of NF-*κ*B ligand
SHH: Sonic hedgehog
SMO: Smoothened
TGF-*β*: Transforming growth factor-*β*
VEGF: Vascular endothelial growth factor.
